# High malnutrition risk burden in the oldest-old in integrated medical and long-term care institutions for older adults: a cross-sectional study

**DOI:** 10.3389/fpubh.2026.1886677

**Published:** 2026-10-29

**Authors:** Jinfeng Gao, Haomou Pu, Yangyang Yan, Li Zhong, Wenqing Zheng, Yan Wu, Xia Zhang, Xiaoping Yu

**Affiliations:** 1 Department of Clinical Nutrition, West China Longquan Hospital Sichuan University (The First People's Hospital of Longquanyi District Chengdu), Chengdu, Sichuan, China; 2 Department of Biomedical Sciences, Faculty of Medicine, University of Macau, Taipa, Macao SAR, China; 3 Department of Clinical Nutrition, Chengdu Jinxin Geriatric Hospital, Chengdu, Sichuan, China; 4 Department of Clinical Nutrition, Chengdu High-tech Jinxin Geriatric Hospital, Chengdu, Sichuan, China; 5 School of Preclinical Medicine, Chengdu University, Chengdu, Sichuan, China

**Keywords:** body mass index, integrated medical and long-term care institutions for older adults, malnutrition risk, mini nutritional assessment–short form, oldest-old

## Abstract

**Objective:**

This study aimed to assess the burden of malnutrition risk among the oldest-old admitted to integrated medical and long-term care institutions for older adults in Chengdu, Sichuan, China, and to evaluate factors associated with malnutrition risk.

**Methods:**

This cross-sectional study included 220 adults aged ≥80 years admitted to three integrated medical and long-term care institutions for older adults between July 2024 and June 2025. Nutritional risk status was assessed using the Mini Nutritional Assessment–Short Form (MNA-SF). Demographic, anthropometric, dietary, laboratory, comorbidity, and functional data were collected. Multivariable logistic regression was used to identify factors associated with malnutrition risk. Restricted cubic splines were used to characterize the shape of the association between BMI and nutritional risk. Because BMI contributes to the MNA-SF score, models including BMI were interpreted as exploratory.

**Results:**

The mean age was 87.46 ± 3.96 years, and 65.5% were women. Overall, 52.7% (95% CI: 46.1–59.4%) had MNA-SF scores in the malnourished range, 44.1% (95% CI: 37.5–50.7%) were at risk of malnutrition, and only 3.2% had a normal nutritional status. In exploratory models that included BMI, higher BMI was associated with lower odds of MNA-SF-defined malnutrition, whether modeled continuously (OR = 0.676, 95% CI: 0.591–0.773), by conventional BMI categories (OR = 0.227, 95% CI: 0.125–0.411), or by older-adult BMI categories (OR = 0.129, 95% CI: 0.062–0.269; all *p* < 0.001). A liquid or semi-liquid diet was associated with higher odds of malnutrition in models with and without BMI (ORs ranging from 2.092 to 2.487, all *p* < 0.05).

**Conclusion:**

Malnutrition risk was highly prevalent among the oldest-old in integrated medical and long-term care institutions for older adults. Routine MNA-SF screening at admission, attention to residents receiving texture-modified diets, and confirmation of the BMI association in independent cohorts may support early nutritional intervention in this vulnerable population.

## 1 Introduction

China is experiencing a rapid demographic transition toward advanced population ageing. According to the seventh national census, adults aged 65 years or older accounted for 13.5% of the Chinese population in 2020 (1). With increasing longevity, multimorbidity, functional impairment, frailty, and complex care needs have become increasingly prominent, particularly among the oldest-old, commonly defined as adults aged 80 years or older (2–4). This population is disproportionately vulnerable to disability, recurrent health-care utilization, and dependence on long-term care services. In response, many health systems have sought to develop integrated care models that connect medical services, rehabilitation, nursing care, and social support (5–7). In China, the integration of health care and long-term care for older adults has been promoted as an important policy and service delivery strategy (8).

Nutritional vulnerability is often an under-recognized component of this care complexity. Malnutrition risk in older adults is rarely an isolated nutritional problem; rather, it is closely intertwined with disease burden, inflammation, anorexia, sarcopenia, cognitive impairment, and functional decline (9). Contemporary consensus statements and clinical guidelines emphasize that malnutrition and malnutrition risk are common in later life and are associated with adverse outcomes, supporting routine screening, timely assessment, and individualized nutritional intervention as essential elements of care for older adults (10). Importantly, malnutrition risk has been linked to higher risks of morbidity, falls, institutionalization, and mortality across clinical contexts (11–13). Therefore, early identification of the level of nutritional risk is particularly important in institutionalized oldest-old populations, and screening instruments are typically the first step in that process.

The Mini Nutritional Assessment–Short Form (MNA-SF) is one of the established screening instruments for older adults and has demonstrated prognostic value in institutional settings, including nursing homes, where lower MNA-SF scores predict subsequent mortality and identify high-risk individuals who may benefit from targeted assessment and intervention (14). In parallel, international diagnostic frameworks such as the Global Leadership Initiative on Malnutrition (GLIM) highlight low body mass index (BMI) as a phenotypic criterion (15). Large cohort analyses suggest that risk of mortality rises at lower BMI values (16), indicating that BMI provides useful information for nutritional assessment and for evaluating health status among older adults. However, BMI alone cannot distinguish sarcopenic obesity from lean mass loss, and its predictive performance may vary across settings, comorbidity profiles, and care models, making setting-specific evidence essential.

Despite growing attention to integrated medical and long-term care for older adults, data remain limited for the oldest-old residing in such institutions, particularly in non-core urban areas where resource allocation, staffing, and nutrition service capacity may differ. Against this background, we conducted a cross-sectional study of adults aged ≥80 years admitted to three integrated medical and long-term care institutions for older adults. We aimed to (i) characterize the level of nutritional risk status at admission using the MNA-SF, (ii) identify factors associated with nutritional risk, and (iii) characterize the shape of the association between BMI and nutritional risk using restricted cubic splines. The findings may inform admission-based nutritional screening, early identification of nutritional risk, and nutrition service planning in these institutions.

## 2 Subjects and methods

### 2.1 Study design and participants

This cross-sectional study was conducted in three integrated medical and long-term care institutions for older adults in Chengdu, Sichuan, China. Integrated medical and long-term care institutions for older adults were defined as facilities that provide both medical services and long-term nursing care for older residents; each of the three participating institutions delivered inpatient medical care together with long-term nursing care and housed more than 200 older residents, so the three sites were comparable in the range of services provided. Participants were recruited using a stratified convenience cluster sampling strategy. Stratification was based on the administrative districts of Chengdu. Within each of the three selected districts, one eligible facility was selected by convenience, and residents admitted during the study period were then recruited by cluster sampling. Because the oldest-old population is geographically dispersed and data availability was limited, fully random sampling was not feasible; the procedure was therefore designed to maximize representativeness within these practical constraints. Eligible participants were newly admitted residents aged 80 years or older who were admitted between July 2024 and June 2025 and who completed nutritional screening and baseline assessment within one week of admission. Participants were excluded if they were younger than 80 years, were receiving palliative end-of-life care, or were completely bedridden and unable to complete the required assessment. Residents who declined participation or did not consent to the collection of biochemical data were also not enrolled.

### 2.2 Sample size estimation

The minimum sample size required for estimating the prevalence of malnutrition was calculated using the following formula: $n=\frac{Z_{\alpha /2}^{2}p(1-p)}{\delta^{2}}$ , where *Z_α/2_* was set at 1.96 for a two-sided 95% confidence level, p was the anticipated prevalence of malnutrition, and δ was the allowable error. Malnutrition prevalence estimates from Chinese settings ranged from 10.0% among community-dwelling adults aged 80 years or older, assessed with the same instrument (17), to 14.67% among hospitalized adults aged over 65 years in a nationwide multicenter survey (18). On this basis, a prevalence of 10% was adopted for sample-size estimation at the design stage, based on the evidence available at that time. The allowable error was set at 0.05, and the minimum required sample size was 139 participants. The final analytic sample included 220 participants (Figure 1), which exceeded this minimum and provided 116 participants with MNA-SF-defined malnutrition, corresponding to more than 10 outcome events per candidate predictor in the multivariable models. Because the sample size was calculated for prevalence estimation rather than for multivariable modeling, and because the number of participants in some strata was small, no subgroup or interaction analyses were performed. Clustering across the three institutions was considered at the recruitment stage: we enrolled newly admitted residents and excluded long-term residents in order to reduce the influence of institution-specific diet provision and care practices. Residual clustering cannot be fully excluded and is noted below as a limitation.

**Figure 1:**
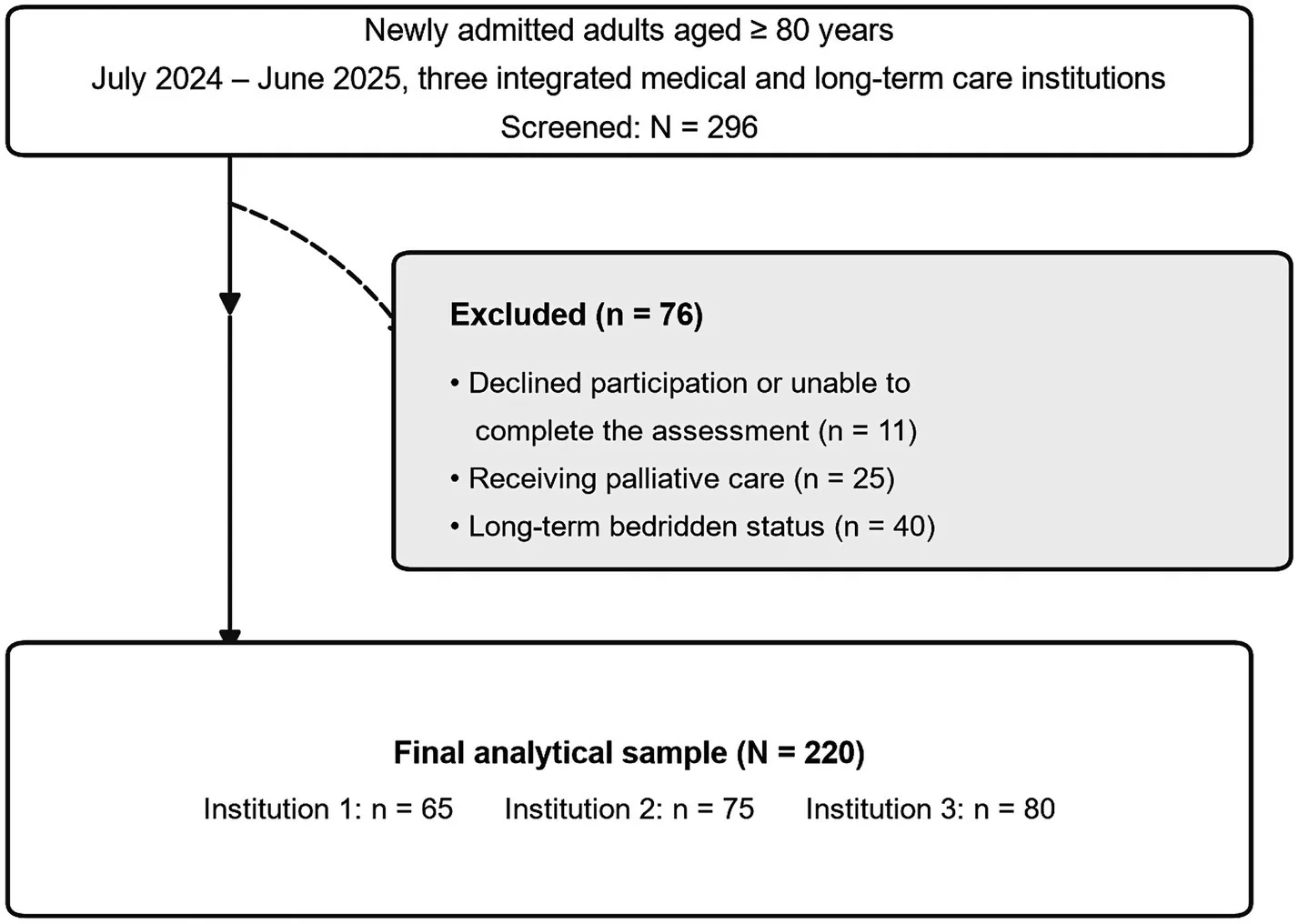
Flowchart of participant recruitment. A total of 296 newly admitted adults aged ≥80 years were screened in three integrated medical and long-term care institutions for older adults between July 2024 and June 2025. Of these, 220 were eligible and enrolled. Seventy-six participants were excluded: 11 declined participation or were unable to complete the assessment, 25 were receiving palliative care, and 40 had long-term bedridden status. The final analytical sample included 220 participants (65 from Institution 1, 75 from Institution 2, and 80 from Institution 3).

### 2.3 Data collection procedures

Data were collected at the time of first admission by trained investigators using a standardized assessment protocol. The level of nutritional risk was assessed using the Mini Nutritional Assessment–Short Form (MNA-SF). Baseline demographic and clinical information was extracted from medical records, including age, sex, marital or partner status, admission information, anthropometric measurements, comorbidities, diet type, functional status, and nutrition-related laboratory indicators. Height and weight were used to calculate body mass index (BMI, kg/m^2^). Body weight was measured at admission by trained investigators using calibrated scales; chair or bed scales were used for participants who could not stand unaided. For participants who could not stand fully upright, height was estimated either by measuring crown-to-heel length in the supine position or from demi-span. Demi-span is the distance from the midline at the sternal notch to the web between the middle and ring fingers along the outstretched arm, and height was derived using the equations of Bassey (19): for women, height in cm = (1.35 × demi-span in cm) + 60.1; for men, height in cm = (1.40 × demi-span in cm) + 57.8. Anemia was defined as a hemoglobin concentration below 120 g/L in men and below 110 g/L in women. The first available hemoglobin and serum albumin measurements after admission were recorded as nutrition-related laboratory indicators. Comorbidities were identified from admission diagnoses and medical records and included seven principal comorbid diagnoses: hypertension, stroke, chronic obstructive pulmonary disease, diabetes, mild cognitive impairment, constipation, and cancer. To obtain consistent baseline data, all laboratory tests, anthropometric measurements, and assessments of diet and functional status were completed within one week of admission.

### 2.4 Measures

The level of nutritional risk was assessed using the Mini Nutritional Assessment–Short Form, a 6-item screening instrument derived from the full Mini Nutritional Assessment and widely used in older populations. The MNA-SF is a screening instrument rather than a diagnostic standard, so the categories below describe screening results and levels of nutritional risk rather than a clinical diagnosis of malnutrition. The MNA-SF evaluates recent food intake, weight loss, mobility, acute disease or psychological stress, neuropsychological problems, and BMI. Total scores range from 0 to 14, with participants categorized as malnourished (serious nutritional risk, 0–7), at risk of malnutrition (8–11), or normal nutritional status (well nourished, no nutritional risk, 12–14) (14, 20). For group comparisons and regression analyses, participants were dichotomized into a malnourished group (MNA-SF score 0–7) and an at-risk group (MNA-SF score 8–11). Because only seven participants had a normal nutritional status, these seven participants were excluded from the dichotomized comparisons and from the primary regression models, and the full sample including them was analyzed as a sensitivity analysis.

BMI was calculated as weight in kilograms divided by height in meters squared. BMI was analyzed both as a continuous variable and as a categorical variable. Two BMI classification schemes were used. First, BMI was categorized according to the conventional Chinese adult criteria as <18.5, 18.5–23.9, and ≥24.0 kg/m^2^ (21). Second, BMI was categorized according to the Chinese health industry standard WS/T 868—2025: appropriate range of body mass index and body weight management standards for the oldest-old as <22.0, 22.0–26.9, and >26.9 kg/m^2^.

Functional status was assessed using the Barthel Index (BI), a 10-item instrument that measures independence in basic activities of daily living (ADL). The assessment covers feeding, bathing, dressing, grooming, toileting, bladder control, bowel control, bed-chair transfer, stair climbing, and ambulation. Total scores range from 0 to 100, with higher scores indicating greater independence (22).

### 2.5 Statistical analysis

Data were managed in Excel 2016 and analyzed using SPSS Statistics (version 25.0) and R (version 4.5.1). Categorical variables are reported as *n* (%), and continuous variables as mean ± standard deviation. Group comparisons of MNA-SF scores were performed using the independent-samples t-test for two-group variables and one-way analysis of variance for variables with three or more groups; comparisons of categorical variables across nutritional risk groups used the chi-square test. Multivariable logistic regression was used to identify factors associated with malnutrition risk. Candidate covariates were selected *a priori* on the basis of clinical knowledge and previous evidence (age, sex, educational level, partner status, serum albumin, anemia, BI score, diet type, and number of comorbidities); variables with *p* < 0.10 in univariable analyses were added to this list rather than replacing it. All 220 participants had complete data for variables included in the models, so complete-case analysis was used and no imputation was required. To examine potential non-linear associations, BMI was modeled using restricted cubic splines with three knots placed at the 10th, 50th, and 90th percentiles of the BMI distribution, using the same covariate adjustment as the primary model and with the reference value set at the median of the observed BMI distribution (20.39 kg/m^2^ in the full sample and 20.21 kg/m^2^ in the sensitivity analysis). Because BMI is one of the six items of the MNA-SF, models that included BMI were interpreted as exploratory and models that excluded BMI were treated as the primary analysis. Given the sample size, no subgroup or interaction analyses were performed. Unless otherwise specified, a two-sided α of 0.05 was considered statistically significant.

## 3 Results

### 3.1 Participant characteristics

A total of 220 adults aged 80 years or older were included in the analysis (Table 1). The mean age was 87.46 ± 3.96 years. Women comprised nearly two-thirds of the study population (65.5%, *n* = 144). Most participants had a middle-school education or below (83.2%, *n* = 183), and three-quarters were widowed or living without a partner (75.9%, *n* = 167). According to the conventional BMI classification, 26.8% of participants were underweight, and 14.1% were overweight or obese. When BMI was categorized according to the Chinese health industry standard WS/T 868—2025, 68.2% had a BMI below 22.0 kg/m^2^, and only 5.5% had a BMI above 26.9 kg/m^2^.

**Table 1:** Demographic and anthropometric characteristics of the study participants.

Characteristic	Category	*n*	%
Age group (years)	80–85	76	34.5
	86–90	78	35.5
	91–95	66	30.0
Sex	Male	76	34.5
	Female	144	65.5
Education level	Middle school or below	183	83.2
	High school/vocational school or above	37	16.8
Widowed/without partner	Yes	167	75.9
	No	53	24.1
BMI (kg/m^2^), scheme 1	<18.5	59	26.8
	18.5–23.9	130	59.1
	≥24.0	31	14.1
BMI (kg/m^2^), scheme 2^*^	<22.0	150	68.2
	22.0–26.9	58	26.4
	>26.9	12	5.5
Total		220	100

BMI, body mass index. *Appropriate range of body mass index and body weight management standards for the oldest-old: WS/T 868—2025.

### 3.2 Nutritional risk status, comorbidity burden, and functional status

The mean MNA-SF score was 7.25 ± 2.23, indicating a generally poor nutritional profile in this oldest-old population (Table 2). Only 7 participants (3.2%) had a normal nutritional status, 97 (44.1%, 95% CI: 37.5–50.7%) were at risk of malnutrition, and 116 (52.7%, 95% CI: 46.1–59.4%) had MNA-SF scores in the malnourished range. Mean MNA-SF scores decreased across nutritional categories, from 12.14 ± 0.38 in participants with a normal nutritional status to 8.93 ± 1.05 in those at risk of malnutrition and 5.54 ± 1.33 in those with MNA-SF-defined malnutrition (*p* < 0.001). Participants receiving a liquid or semi-liquid diet had lower MNA-SF scores than those receiving a regular diet (6.65 ± 2.12 vs. 7.44 ± 2.24, *p* = 0.023). Lower scores were also observed among participants with a low serum albumin level (6.07 ± 2.55, *p* = 0.002) and those with anemia (6.82 ± 2.21, *p* = 0.037).

**Table 2:** Nutritional risk status, nutrition-related clinical characteristics, comorbidity burden, and functional status of the study participants.

Variable	Category	*n* (%)	MNA-SF score (mean ± SD)	*t*/*F*	*p*
MNA-SF category	Normal nutritional status	7 (3.2)	12.14 ± 0.38	270.648	<0.001
	At risk of malnutrition	97 (44.1)	8.93 ± 1.05		
	Malnourished	116 (52.7)	5.54 ± 1.33		
Diet type	Regular diet	165 (75.0)	7.44 ± 2.24	2.289	0.023
	Liquid/semi-liquid	55 (25.0)	6.65 ± 2.12		
Nutrition-related diseases	serum albumin <35 g/L	30 (13.6)	6.07 ± 2.55	−3.177	0.002
	anemia*	77 (35.0)	6.82 ± 2.21	−2.100	0.037
Number of comorbidities**	≤3	159 (72.3)	7.04 ± 2.26	−2.249	0.025
	≥4	61 (27.7)	7.79 ± 2.08		
BI score	0–40	175 (79.5)	7.18 ± 2.10	5.637	0.004
	41–60	30 (13.6)	6.77 ± 2.73		
	61–99	15 (6.8)	9.00 ± 2.00		
Total		220 (100)	7.25 ± 2.23		

BI, Barthel Index; MNA-SF, Mini Nutritional Assessment–Short Form; SD, standard deviation. *anemia was defined as hemoglobin <120 g/L in men and <110 g/L in women. **Comorbidities included hypertension, stroke, chronic obstructive pulmonary disease, diabetes, mild cognitive impairment, constipation and cancer.

A total of 72.3% of participants had three or fewer comorbidities, and 27.7% had four or more comorbidities. Hypertension, stroke, and chronic obstructive pulmonary disease were the most common diagnoses, while constipation was present in 29.5% of participants. Functional dependence was also prominent. 79.5% of participants had a BI score of 0–40.

### 3.3 Group comparisons according to MNA-SF category

Participants were classified into a malnourished group (MNA-SF score 0–7; *n* = 116) and a risk of malnutrition group (MNA-SF score 8–11; *n* = 97). BMI distribution differed significantly between the two groups under both classification schemes (Table 3). Using the conventional BMI classification, MNA-SF-defined malnutrition was most prevalent among participants with BMI < 18.5 kg/m^2^, affecting 79.7% of this subgroup (*p* < 0.001). A similar pattern was observed when BMI was categorized according to the Chinese health industry standard for older adults. 68.0% of participants with BMI < 22.0 kg/m^2^ were malnourished, and the corresponding proportions were 24.1 and 11.1% among those with BMI 22.0–26.9 kg/m^2^ and >26.9 kg/m^2^, respectively (*p* < 0.001). In addition, the proportion of participants with MNA-SF-defined malnutrition was higher among those with a partner (66.7%) than among those widowed or without a partner (50.6%, *p* = 0.045).

**Table 3:** Comparison of participant characteristics according to MNA-SF category.

Variable	Category	*n*	MNA-SF category		Test statistic	*p*
			Malnourished *n* (%)/rank	Malnutrition risk *n* (%)/rank		
Age group (years)	80–85	74	34 (45.9)	40 (54.1)	χ^2^ = 3.674	0.159
	86–90	75	46 (61.3)	29 (38.7)		
	91–95	64	36 (56.3)	28 (43.7)		
Sex	Male	75	46 (61.3)	29 (38.7)	χ^2^ = 2.205	0.138
	Female	138	70 (50.7)	68 (49.3)		
Education level	Middle school or below	179	95 (53.1)	84 (46.9)	χ^2^ = 0.870	0.351
	High school/vocational or above	34	21 (61.8)	13 (38.2)		
Widowed/without partner	Yes	162	82 (50.6)	80 (49.4)	χ^2^ = 4.029	0.045
	No	51	34 (66.7)	17 (33.3)		
BMI (kg/m^2^), scheme 1	<18.5	59	47 (79.7)	12 (20.3)	χ^2^ = 29.146	<0.001
	18.5–23.9	128	64 (50.0)	64 (50.0)		
	≥24.0	26	5 (19.2)	21 (80.8)		
BMI (kg/m^2^), scheme 2^*^	<22.0	150	102 (68.0)	48 (32.0)	χ^2^ = 38.011	<0.001
	22.0–26.9	54	13 (24.1)	41 (75.9)		
	>26.9	9	1 (11.1)	8 (88.9)		
Diet type	Regular diet	160	81 (50.6)	79 (49.4)	χ^2^ = 3.813	0.051
	Liquid/semi-liquid	53	35 (66.0)	18 (34.0)		
Anemia**	Yes	77	45 (58.4)	32 (41.6)	χ^2^ = 0.771	0.380
	No	136	71 (52.2)	65 (47.8)		
Number of comorbidities***	0–3	155	90 (58.1)	65 (41.9)	χ^2^ = 2.982	0.084
	≥4	58	26 (44.8)	32 (55.2)		
Serum albumin (g/L)	<35	30	20 (66.7)	10 (33.3)	χ^2^ = 2.098	0.148
	≥35	183	96 (52.5)	87 (47.5)		
BI score	0–40	171	95 (55.6)	76 (44.4)	χ^2^ = 1.430	0.489
	41–60	29	16 (55.2)	13 (44.8)		
	61–99	13	5 (38.5)	8 (61.5)		

BMI, body mass index; BI, Barthel Index; MNA-SF, Mini Nutritional Assessment–Short Form. The seven participants with a normal nutritional status (MNA-SF score 12–14) were excluded, leaving 213 participants; values are *n* (%) within each row category, and group comparisons used the chi-square test. *BMI categories were defined according to the recommended BMI range for older adults in the Chinese health industry standard WS/T 868—2025. **Anemia was defined as hemoglobin <120 g/L in men and <110 g/L in women. ***Comorbidities included hypertension, stroke, chronic obstructive pulmonary disease, diabetes, mild cognitive impairment, constipation, and cancer.

### 3.4 Multivariable analysis of factors associated with nutritional risk

Nutritional risk status defined by the MNA-SF was used as the dependent variable in the logistic regression models. Candidate covariates were selected *a priori* on clinical grounds and supplemented by variables with *p* < 0.10 in univariable analyses. The final models included age, sex, educational level, partner status, serum albumin, anemia, BI score, diet type, and number of comorbidities (Tables 4–8). Because only seven participants had a normal nutritional status, the primary models excluded these participants and compared MNA-SF-defined malnutrition (score 0–7) with risk of malnutrition (score 8–11); a sensitivity analysis in the full sample of 220 participants, in which the seven participants with a normal nutritional status were grouped with those at risk, gave materially similar estimates for the principal predictors (Tables 4, 5). Collinearity was assessed using variance inflation factors for the continuous covariates (age and BMI); all values were below 2, indicating no material multicollinearity. The Hosmer–Lemeshow test indicated adequate fit for the primary model (χ^2^ = 5.188, *p* = 0.737) and for the other specifications, including the full-sample analysis, the analysis excluding the seven well-nourished participants, and the analysis without BMI (all *p* > 0.05). Cook’s distances ranged from 0.007 to 0.260, all far below the conventional threshold of 1, indicating no influential observations.

**Table 4:** Multivariable logistic regression models for malnutrition defined by MNA-SF category (Model 1).

Predictor	*β*	Wald *χ^2^*	*P*	OR	95% CI
Age^a^	0.067	3.273	0.070	1.069	(0.994–1.150)
Sex^b^	−0.350	1.190	0.275	0.705	(0.376–1.321)
Education level^b^	0.386	0.950	0.330	1.471	(0.677–3.195)
Widowed/without partner^b^	−0.642	3.226	0.072	0.526	(0.261–1.060)
Diet type^b^	0.738	4.536	0.033	2.092	(1.061–4.125)
Number of comorbidities^b^	−0.757	5.261	0.022	0.469	(0.245–0.896)
BI score^c^	−0.331	1.764	0.184	0.718	(0.440–1.171)
Serum albumin (g/L)^b^	−0.475	1.136	0.286	0.622	(0.260–1.489)
Anemia^b^	0.241	0.577	0.447	1.273	(0.683–2.371)

Model 1 included all 220 participants (116 with MNA-SF-defined malnutrition and 104 without). Reference categories were male for sex, middle school or below for educational level, having a partner for widowed/without partner, regular diet for diet type, fewer than four for number of comorbidities, and no anemia for anemia. 𝐼ontinuous variable; the odds ratio represents the change in odds per 1-unit increase. 𝑻inary variable; the odds ratio compares the named category with the reference category. ᶜOrdinal variable; the odds ratio represents the change in odds per 1-category increase (BI score categories 0–40, 41–60, and 61–99).

**Table 5:** Multivariable logistic regression models for malnutrition defined by MNA-SF category (Model 2).

Predictor	*β*	Wald *χ*^2^	*p*	OR	95% CI
Age^a^	0.066	3.047	0.081	1.068	(0.992–1.150)
Sex^b^	−0.286	0.780	0.377	0.751	(0.398–1.418)
Education level^b^	0.529	1.642	0.200	1.698	(0.756–3.815)
Widowed/without partner^b^	−0.718	3.788	0.052	0.488	(0.237–1.005)
Diet type^b^	0.799	5.058	0.025	2.223	(1.108–4.459)
Number of comorbidities^b^	−0.683	4.142	0.042	0.505	(0.262–0.975)
BI score^c^	−0.225	0.756	0.384	0.799	(0.482–1.325)
Serum albumin (g/L)^b^	−0.426	0.913	0.339	0.653	(0.273–1.565)
Anemia^b^	0.147	0.211	0.646	1.158	(0.619–2.170)

Model 2 excluded the seven participants with a normal nutritional status (MNA-SF score 12–14), leaving 213 participants (116 with MNA-SF-defined malnutrition and 97 at risk). 𝐼ontinuous variable; the odds ratio represents the change in odds per 1-unit increase. 𝑻inary variable; the odds ratio compares the named category with the reference category. ᶜOrdinal variable; the odds ratio represents the change in odds per 1-category increase (BI score categories 0–40, 41–60, and 61–99).

**Table 6:** Multivariable logistic regression models for malnutrition defined by MNA-SF category (Model 3).

Predictor	*β*	Wald *χ*^2^	*p*	OR	95% CI
Age^a^	0.055	1.632	0.201	1.056	(0.971–1.148)
Sex^b^	−0.481	1.657	0.198	0.618	(0.297–1.286)
Education level^b^	0.404	0.758	0.384	1.498	(0.603–3.719)
Widowed/without partner^b^	−1.152	7.051	0.008	0.316	(0.135–0.739)
Diet type^b^	0.911	5.195	0.023	2.487	(1.136–5.445)
Number of comorbidities^b^	−0.623	2.765	0.096	0.537	(0.258–1.118)
BI score^c^	−0.048	0.028	0.866	0.953	(0.544–1.670)
Serum albumin (g/L)^b^	−0.867	2.535	0.111	0.420	(0.145–1.222)
Anemia^b^	−0.246	0.433	0.511	0.782	(0.375–1.629)
BMI^a,*^	−0.391	32.700	<0.001	0.676	(0.591–0.773)

*Model 3 additionally included BMI as a continuous variable and was based on the 213 participants retained in Model 2. Because BMI is one of the six MNA-SF items, this model was interpreted as exploratory. 𝐼ontinuous variable; the odds ratio represents the change in odds per 1-unit increase. 𝑻inary variable; the odds ratio compares the named category with the reference category. ᶜOrdinal variable; the odds ratio represents the change in odds per 1-category increase (BI score categories 0–40, 41–60, and 61–99).

**Table 7:** Multivariable logistic regression models for malnutrition defined by MNA-SF category (Model 4).

Predictor	*β*	Wald *χ^2^*	*P*	OR	95% CI
Age^a^	0.053	1.694	0.193	1.054	(0.974–1.142)
Sex^b^	−0.277	0.629	0.428	0.758	(0.383-1.502)
Education level^b^	0.402	0.850	0.357	1.495	(0.636–3.513)
Widowed/without partner^b^	−0.909	5.104	0.024	0.403	(0.183–0.887)
Diet type^b^	0.852	4.933	0.026	2.344	(1.105–4.969)
Number of comorbidities^b^	−0.590	2.755	0.097	0.554	(0.276–1.113)
BI score^c^	−0.108	0.152	0.697	0.898	(0.552–1.543)
Serum albumin (g/L)^b^	−0.818	2.559	0.110	0.441	(0.162–1.202)
Anemia^b^	−0.155	0.193	0.661	0.857	(0.429–1.709)
BMI^c,*^	−1.485	23.883	<0.001	0.227	(0.125–0.411)

*Model 4 additionally included BMI, entered as an ordinal variable across the three conventional categories (<18.5, 18.5–23.9, and ≥24.0 kg/m^2^). A single odds ratio is therefore reported for BMI, representing the change in odds per one-category increase rather than a comparison of a specific category with a reference category. 𝐼ontinuous variable; the odds ratio represents the change in odds per 1-unit increase. 𝑻inary variable; the odds ratio compares the named category with the reference category. ᶜOrdinal variable; the odds ratio represents the change in odds per 1-category increase (BI score categories 0–40, 41–60, and 61–99).

**Table 8:** Multivariable logistic regression models for malnutrition defined by MNA-SF category (Model 5).

Predictor	*β*	Wald *χ*^2^	*p*	OR	95% CI
Age^a^	0.038	0.805	0.370	1.038	(0.956–1.127)
Sex^b^	−0.626	2.809	0.094	0.535	(0.257–1.112)
Education level^b^	0.397	0.780	0.377	1.488	(0.616–3.592)
Widowed/without partner^b^	−0.940	4.999	0.025	0.391	(0.171–0.890)
Diet type^b^	0.814	4.366	0.037	2.258	(1.052–4.848)
Number of comorbidities^b^	−0.654	3.253	0.071	0.520	(0.255–1.058)
BI score^c^	−0.196	0.462	0.497	0.822	(0.468–1.446)
Serum albumin (g/L)^b^	−0.796	2.359	0.125	0.451	(0.163–1.246)
Anemia^b^	−0.030	0.007	0.934	0.970	(0.479–1.966)
BMI^c,*^	−2.049	29.841	<0.001	0.129	(0.062–0.269)

*Model 5 additionally included BMI, entered as an ordinal variable across the three categories of the Chinese health industry standard WS/T 868—2025 (<22.0, 22.0–26.9, and >26.9 kg/m^2^). A single odds ratio is therefore reported for BMI, representing the change in odds per one-category increase rather than a comparison of a specific category with a reference category. 𝐼ontinuous variable; the odds ratio represents the change in odds per 1-unit increase. 𝑻inary variable; the odds ratio compares the named category with the reference category. ᶜOrdinal variable; the odds ratio represents the change in odds per 1-category increase (BI score categories 0–40, 41–60, and 61–99).

After adjustment, a liquid or semi-liquid diet was associated with higher odds of malnutrition (OR = 2.092, 95% CI: 1.061–4.125, *p* = 0.033 in the full sample; OR = 2.223, 95% CI: 1.108–4.459, *p* = 0.025 after exclusion of the seven participants with a normal nutritional status), whereas having four or more comorbidities was associated with lower odds (OR = 0.469, 95% CI: 0.245–0.896, *p* = 0.022; and OR = 0.505, 95% CI: 0.262–0.975, *p* = 0.042, respectively).

Because BMI is one of the six MNA-SF items, models that included BMI were interpreted as exploratory. The BMI item of the MNA-SF is scored using cut-off values of 19, 21, and 23 kg/m^2^, and categorical BMI was additionally defined according to the recommended range for older adults in the Chinese health industry standard WS/T 868—2025. In these exploratory models, BMI was entered as an ordinal variable across the three ordered categories, so a single odds ratio is reported for each classification scheme and represents the change in odds per one-category increase rather than a comparison of a specific category with a reference category. Higher BMI was associated with lower odds of malnutrition (Tables 6–8). When BMI was treated as a continuous variable, each 1-kg/m^2^ increase was associated with lower odds of malnutrition (OR = 0.676, 95% CI: 0.591–0.773; *p* < 0.001). Similar associations were observed when BMI was categorized using the conventional classification scheme (OR = 0.227, 95% CI: 0.125–0.411; *p* < 0.001) or the Chinese health industry standard for older adults (OR = 0.129, 95% CI: 0.062–0.269; *p* < 0.001). These estimates quantify the strength of association in this cross-sectional sample and should not be read as evidence of the predictive or discriminatory performance of BMI.

Diet type was also associated with malnutrition after adjustment for BMI and other confounding factors. Compared with participants receiving a regular diet, those receiving a liquid or semi-liquid diet had approximately two-fold higher odds of malnutrition across the models, with odds ratios ranging from 2.258 to 2.487 (all *p* < 0.05). In Models 3, 4 and 5, being widowed or living without a partner was associated with lower odds of malnutrition after adjustment for diet type and BMI (OR = 0.316, 95% CI: 0.135–0.739, *p* = 0.008; OR = 0.403, 95% CI: 0.183–0.887, *p* = 0.024; OR = 0.391, 95% CI: 0.171–0.890, *p* = 0.025), although this association was not statistically significant in Models 1 and 2. Because diet texture is usually prescribed in response to underlying clinical conditions, the diet-type association is likely to reflect confounding by indication rather than an independent effect.

### 3.5 Restricted cubic spline analysis of BMI and nutritional risk

Restricted cubic spline analysis was used to characterize the shape of the association between BMI and malnutrition risk (Figure 2). After adjustment for age, sex, educational level, partner status, serum albumin, anemia, BI score, diet type, and number of comorbidities, BMI showed a significant overall association with malnutrition risk (*P* for overall association < 0.001). The odds of malnutrition decreased progressively with increasing BMI, with no significant departure from linearity (*P* for non-linearity = 0.576); each 1-unit increment in BMI was associated with approximately 33.4% lower odds of malnutrition (OR = 0.666). Confidence intervals are wide at both extremes of the distribution, where few participants contributed information. Twenty-one participants had a BMI below 16 kg/m^2^ and 9 had a BMI above 28 kg/m^2^. Because BMI is one of the six MNA-SF items, the curve should be interpreted as descriptive of association rather than as evidence of discriminative performance. Exclusion of the seven participants with a normal nutritional status gave a similar curve (Figure 3; *p* for non-linearity = 0.662), with each 1-unit increment in BMI associated with approximately 32.4% lower odds (OR = 0.676).

**Figure 2:**
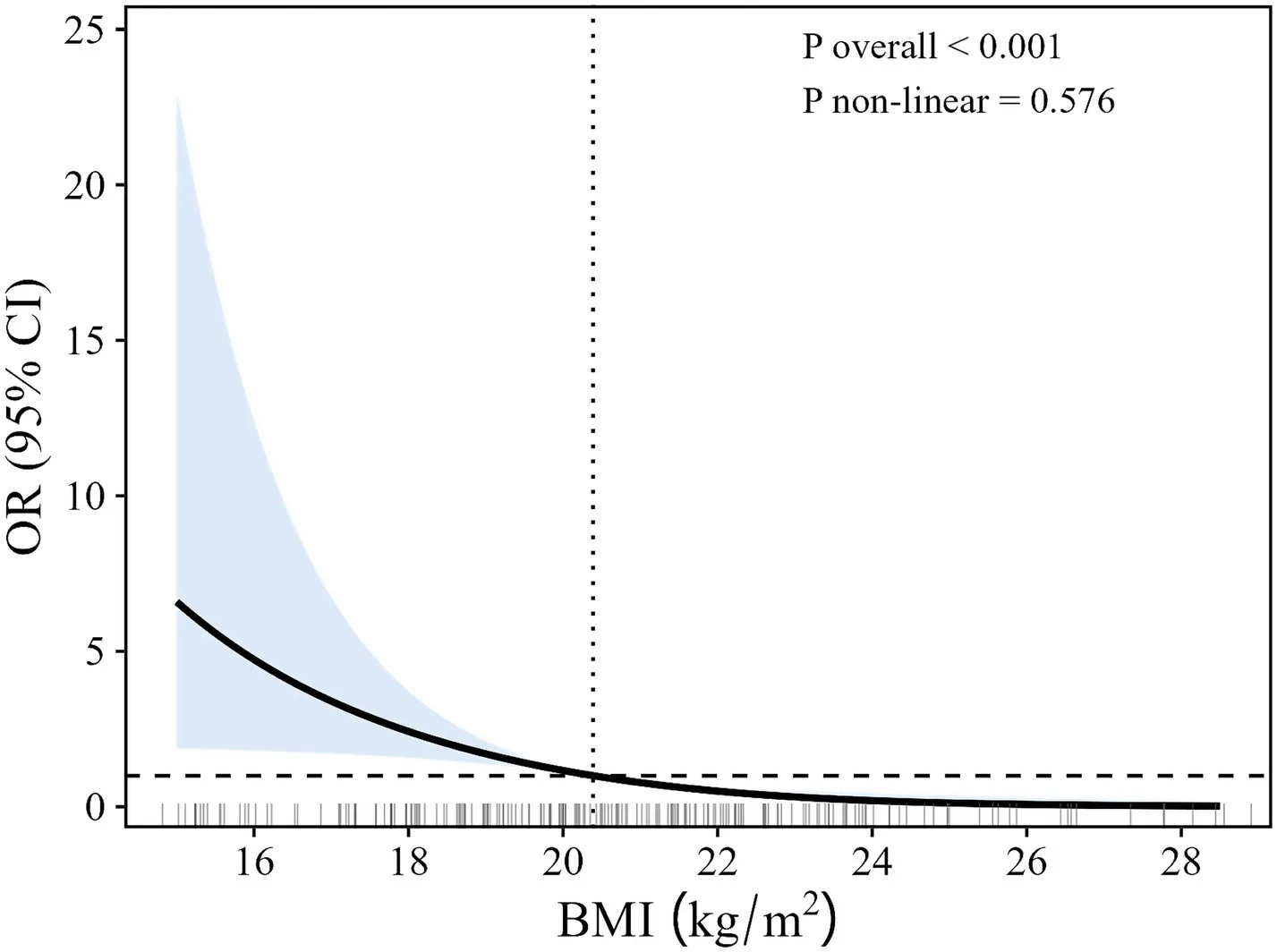
Restricted cubic spline association between BMI and malnutrition risk among all 220 participants. The model was fitted with three knots placed at the 10th, 50th, and 90th percentiles of the BMI distribution and was adjusted for age, sex, educational level, partner status, serum albumin, anemia, BI score, diet type, and number of comorbidities. Odds ratios (ORs) and 95% confidence intervals (shaded area) for malnutrition risk are shown. The dotted lines denote the reference values (BMI = 20.39 kg/m^2^, the median of the observed BMI distribution; OR = 1.0); the reference value was therefore distribution-based rather than derived from a threshold identified in these data.

**Figure 3:**
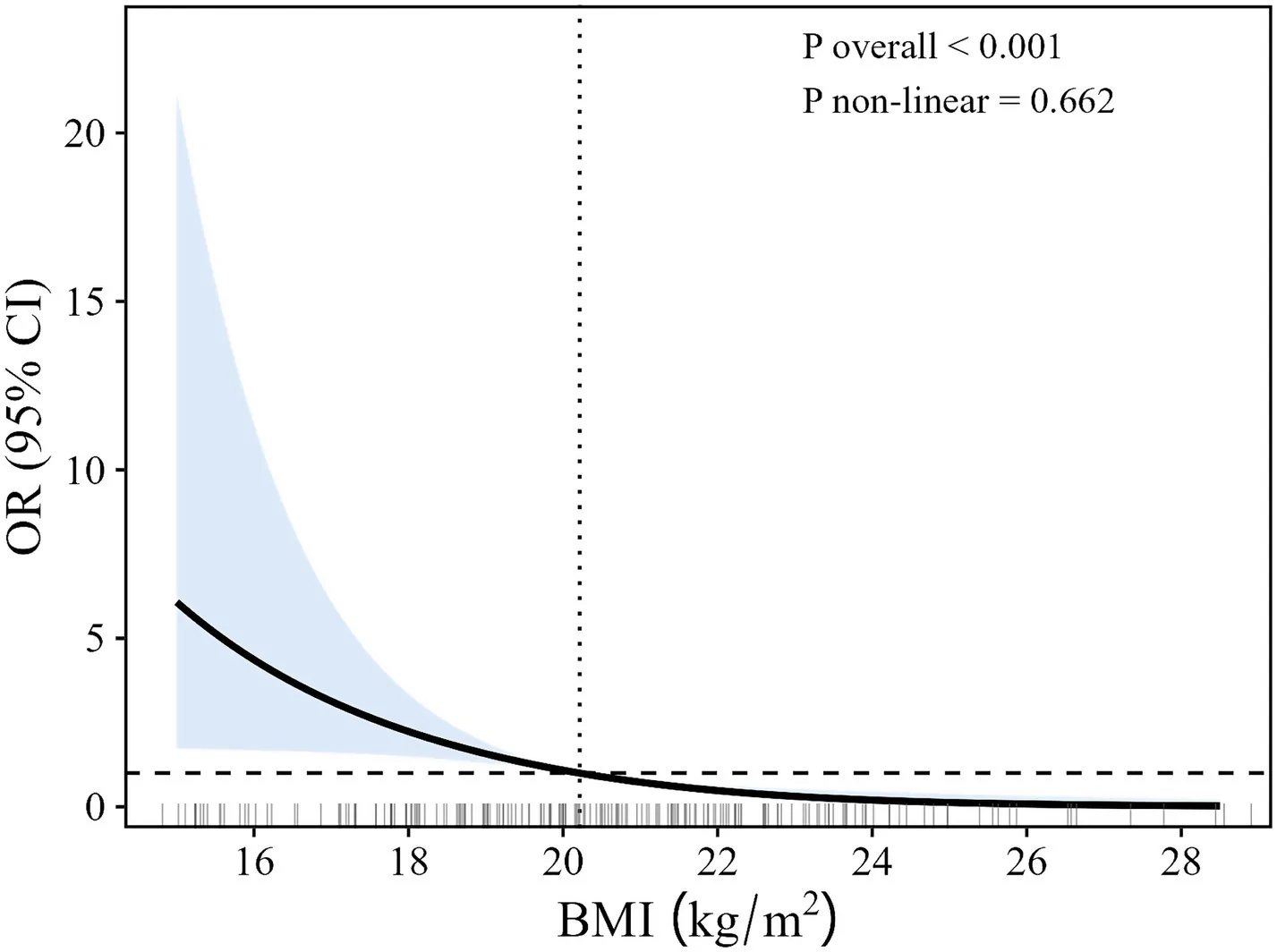
Restricted cubic spline association between *BMI* and malnutrition risk after exclusion of the seven participants with a normal nutritional status. The model was fitted with three knots placed at the 10th, 50th, and 90th percentiles of the *BMI* distribution and was adjusted for the same covariates as Figure 2. Odds ratios (*ORs*) and 95% confidence intervals (shaded area) for malnutrition risk are shown. The dotted lines denote the reference values (*BMI* = 20.21 kg/m2, the median of the observed *BMI* distribution in this subsample; *OR* = 1.0).

## 4 Discussion

In this cross-sectional study of 220 adults aged 80 years or older admitted to integrated medical and long-term care institutions for older adults in Chengdu, Sichuan, more than half of the participants had MNA-SF scores in the malnourished range, and almost all of the remaining participants were at risk of malnutrition. Only 3.2% had a normal nutritional status. The malnutrition risk burden observed in this study was considerably higher than that reported in community-based older populations. A community study from Southwest China using the MNA-SF reported that 3.8% of older adults were malnourished and 39.2% were at risk of malnutrition, although the burden increased markedly among individuals aged 80 years or older, with 10.0% malnourished and 64.0% at risk of malnutrition (17). The present study found a much higher proportion of participants with MNA-SF-defined malnutrition, despite using the same age threshold of ≥80 years and the same instrument. This discrepancy is likely attributable to differences in care setting, disease burden, functional dependence, and admission status. Residents often represent a transitional population between community-dwelling older adults and hospitalized or long-term care patients. They are more likely to have multimorbidity, recent functional decline, dietary modification, and higher care dependency, all of which may accelerate nutritional deterioration (23–26).

A multinational pooled analysis using the Mini Nutritional Assessment reported an overall malnutrition prevalence of 22.8% among older adults, but prevalence varied widely by setting, being lower in community-dwelling populations and higher in hospitals, rehabilitation facilities, and institutional care settings (27). A later meta-analysis similarly showed that malnutrition prevalence was lowest in community settings and substantially higher in hospitals, nursing homes, long-term care, and rehabilitation or subacute care settings (28). The very high prevalence in our study therefore does not contradict previous literature. It highlights the particularly vulnerable nutritional profile of the oldest-old population entering integrated medical and long-term care institutions for older adults.

A further finding of this study was the inverse association between BMI and MNA-SF-defined malnutrition; each 1-kg/m^2^ increase in BMI was associated with approximately 33% lower odds. Because BMI is one of the six MNA-SF items, part of this association is built into the structure of the instrument, and the estimates describe association rather than predictive performance. The restricted cubic spline analysis provided additional support by showing a significant overall association without evidence of non-linearity, suggesting that within the observed BMI range, malnutrition risk declined progressively as BMI increased. Previous Chinese inpatient research has reported correlations between MNA, MNA-SF, BMI, serum albumin, hemoglobin, and other anthropometric or biochemical indicators, supporting the clinical relevance of BMI as a simple marker of nutritional status in older Chinese populations (29). However, muscle status was not assessed among participants in this study; the GLIM criteria for malnutrition diagnosis could not be applied, which was regarded as a limitation of the present study (30). Only hemoglobin and serum albumin were available as nutrition-related laboratory indicators here, and both are non-specific and are influenced by inflammation, hydration, and chronic disease. Routinely available biochemical, hematological, and immunological parameters have been evaluated for identifying malnutrition in hospitalized older patients (31), and oxidative stress parameters and adipokines have been examined as candidate biomarkers linking nutritional impairment to broader vulnerability in older adults (32). The absence of these more specific markers limits the biochemical characterization of nutritional risk in the present study. In very old adults, body composition changes, sarcopenia, edema, and height measurement error may weaken the precision of BMI. Nevertheless, because BMI is inexpensive, rapid, and operationally feasible, it may still be useful as a first indicator of nutritional risk in resource-limited institutional settings, pending validation in independent cohorts.

Diet type was another factor associated with malnutrition. Participants receiving liquid or semi-liquid diets had approximately two-fold higher odds of malnutrition than those receiving regular diets. This association may reflect several overlapping mechanisms. Liquid or semi-liquid diets are often prescribed for residents with dysphagia, poor dentition, neurological disease, gastrointestinal symptoms, or acute illness, all of which may reduce energy and protein intake. Texture-modified diets can have lower energy density and reduced palatability if not carefully fortified. Moreover, diet modification may also be a marker of frailty or disease severity rather than a direct cause of malnutrition. ESPEN guidance emphasizes that older persons with malnutrition or at risk of malnutrition in institutional settings should receive structured nutrition care, adequate resources, and individualized support for food and fluid intake (33). Our findings support the need for systematic nutritional monitoring among residents receiving texture-modified diets, particularly at admission. Because swallowing status and the indications for diet modification were not recorded, this association is likely to reflect confounding by indication and possible reverse causation rather than an independent causal effect of food texture.

Partner status was also associated with MNA-SF-defined malnutrition in the exploratory models that included BMI, although the association was not consistent across models and was not statistically significant in the models that excluded BMI. This observation is preliminary, and larger multicenter studies should examine whether social support modifies nutritional risk in institutionalized oldest-old populations (34).

This study has several practical implications. First, nutritional screening at admission should be routine in integrated medical and long-term care institutions for older adults, especially for residents aged 80 years or older. Second, liquid or semi-liquid diet should be treated as a warning signal requiring dietitian review, energy and protein enrichment, swallowing evaluation when indicated, and close monitoring of intake. Third, nutrition management should be integrated with functional rehabilitation and nursing care, because malnutrition, disability, and multimorbidity are tightly interconnected in the oldest-old. The study focused specifically on the oldest-old population, a group often underrepresented in nutritional epidemiology despite their high clinical vulnerability. Data were collected at first admission, allowing early identification of nutritional risk before prolonged institutional care could further modify nutritional status. The study also evaluated BMI using both conventional and age-specific Chinese BMI classifications and applied restricted cubic splines to characterize the shape of the BMI association, while additionally reporting models that excluded BMI in order to address the overlap between BMI and the MNA-SF.

Several limitations should also be acknowledged. First, BMI is one of the six items of the MNA-SF, so using BMI as a predictor of MNA-SF-defined nutritional risk introduces incorporation bias. The association observed between BMI and nutritional risk is therefore partly determined by the structure of the instrument and should not be interpreted as independent predictive or discriminatory performance of BMI. We addressed this by reporting models that excluded BMI as the primary analysis (Models 1 and 2) and by treating all models containing BMI as exploratory; an outcome definition fully independent of BMI could not be constructed from the available data. This limitation is distinct from the more general concern that BMI is an imperfect measure of body composition. Second, because of the cross-sectional design, the observed associations between BMI, diet type, and nutritional risk may be bidirectional, and no temporal or causal inference is possible. Third, participants were recruited from three institutions in Chengdu, Sichuan, using a stratified convenience cluster sampling strategy, and clustering across institutions was not modeled; this may limit generalizability to other regions or to community-dwelling older adults. Fourth, completely bedbound residents and those receiving palliative end-of-life care were excluded because they could not complete the MNA-SF assessment. Because these individuals are likely to have even higher nutritional risk and greater functional dependence, the true burden of malnutrition may have been underestimated, and the observed associations with functional status and diet type may have been attenuated; the findings should therefore not be extrapolated to this group. Fifth, dietary intake, inflammatory markers, swallowing function, oral health, sarcopenia, and detailed body composition were not available, limiting mechanistic interpretation (31, 32). Finally, all estimates were obtained in a single sample of 220 participants without internal or external validation and require confirmation in larger multicenter cohorts.

## 5 Conclusion

This study identified a substantial burden of malnutrition risk among adults aged 80 years or older admitted to integrated medical and long-term care institutions for older adults in Chengdu, Sichuan, China. Higher BMI was associated with lower odds of malnutrition in an approximately linear dose–response manner, but because BMI contributes to the MNA-SF score, this association is exploratory and requires confirmation in independent cohorts. Texture-modified diets and partner status should be considered when assessing nutritional risk among the oldest-old, while recognizing that diet texture is usually prescribed in response to underlying clinical conditions. These findings support routine MNA-SF screening at admission, age-appropriate interpretation of BMI, and targeted nutritional care for residents with low BMI and texture-modified diets.

## Data Availability

The raw data supporting the conclusions of this article will be made available by the authors, without undue reservation.
